# Calcitriol and hyperthermia potentiate gemcitabine efficacy – A Multifactorial Preclinical Evaluation in Pancreatic and Breast Cancer

**DOI:** 10.21203/rs.3.rs-7593235/v1

**Published:** 2025-10-23

**Authors:** Olga Wiecheć-Cudak, Aleksandra Murzyn, Gniewosz Drwięga, Aleksandra Bienia, Adam Kłóś, Dominik Robak, Krystyna Urbańska, Ivana Stanimirova-Daszykowska, Andrzej T. Słomiński, Martyna Elas, Martyna Krzykawska-Serda

**Affiliations:** Jagiellonian University; Jagiellonian University; Jagiellonian University; Jagiellonian University; Jagiellonian University; Jagiellonian University; Jagiellonian University; University of Silesia; University of Alabama at Birmingham; Jagiellonian University; Jagiellonian University

**Keywords:** Combination therapy, Gemcitabine, Calcitriol, Hyperthermia, Multimodal treatment, PDAC mouse model

## Abstract

Pancreatic ductal adenocarcinoma (PDAC) remains one of the most lethal cancers, characterized by chemoresistance and poor prognosis. Combination therapies that target multiple tumor vulnerabilities simultaneously are a promising strategy to overcome treatment limitations. This study aimed to evaluate the therapeutic potential and synergistic effects of a triple-modality treatment comprising gemcitabine (GEM), calcitriol (CAL), and hyperthermia (HT) in preclinical models of pancreatic and breast cancer.

In vitro experiments were conducted using three cancer cell lines: human PDAC (PANC-1), murine PDAC (Panc02), and murine triple-negative breast cancer (4T1). Treatments included gemcitabine (GEM), calcitriol (CAL), and hyperthermia (HT). Cell viability, apoptosis, and metabolic activity were assessed via cell counting, MTT assays, real-time live cell imaging, and flow cytometry. Protein expression of VDR and HSP70 was analyzed by Western blotting. In vivo, Panc02 tumors were orthotopically implanted in C57BL/6J mice and treated with GEM, CAL and HT. Tumor growth was monitored by ultrasound, and survival was evaluated using Cox regression models.

Triple therapy significantly reduced cell viability and metabolic activity across all models, with the strongest cytotoxic effects observed in 4T1 cells. Synergistic effects were observed at low GEM concentrations, especially in resistant PANC-1 cells. In vivo, triple therapy inhibited tumor growth, reduced peritoneal metastases, and improved survival (~ 85%), with limited systemic toxicity.

The combination of GEM, CAL, and HT shows strong synergistic anti-cancer effects both in vitro and in vivo. This triple therapy enhances treatment outcomes in resistant tumors such as PDAC and represents a clinically relevant, low-toxicity approach for multimodal cancer treatment.

## Introduction

Combination therapy, i.e. use of two or more therapeutic factors, is more effective than monotherapy because it targets key pathways in a synergistic or additive manner [1]. Additionally, the combination therapy can prevent toxic effects on normal cells, while showing anti-cancer effects [2] and may serve as a useful strategy to counteract the ability of advanced cancers to regulate local and systemic homeostasis [3].

**Fig. 1.**
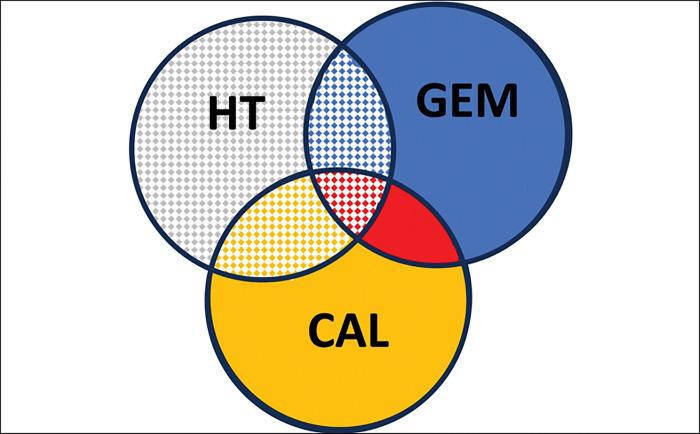
Combination therapy treatment groups scheme. Three therapeutic modalities included gemcitabine (GEM, blue), calcitriol (CAL, yellow), and hyperthermia (HT, regardless of the color, always dashed). Vehicle-treated control cells/mice (saline, grey color). Other experimental groups were two-factor combinations: GEM+CAL (red), GEM+HT (blue dashed), HT+CAL (yellow dashed line). Triple-factor treatment is shown in red dashed. The same color coding is used throughout the article.

In this study, three different treatment components, such as the anti-cancer drug gemcitabine, calcitriol [1,25 (dihydroxyvitamin D3 (1,25(OH)_2_D_3_], and hyperthermia (Fig. 1), were used. Gemcitabine (Difluorodeoxycytidine, dFdC), the antimetabolite, is a specific analogue of deoxycytidine affecting the DNA synthesis process. After gemcitabine is incorporated into the DNA strand, only one more nucleotide can be added, halting DNA polymerase activity. This incorporation of phosphorylated gemcitabine disrupts DNA synthesis and leads to cell death. In some cancer cells, e.g. pancreatic cancer, gemcitabine induces apoptosis by activating caspases, e.g. caspase 3 [4]. Also, some genes regulating apoptosis, such as p53, bcl-2, bcl-xL and BAX, may have a significant influence on gemcitabine sensitivity in cells [5].

Hyperthermia is a targeted cancer therapy that raises tissue temperature and is typically used alongside chemotherapy or radiotherapy to enhance treatment effectiveness. When combined with chemotherapy, hyperthermia sensitizes cancer cells and enhances drug accumulation and anti-tumor efficacy [6]. Hyperthermia’s assumed mechanisms include induction of heat shock proteins (HSPs), regulation of apoptosis, modulation of drug resistance, and altered signal transduction pathways. Hyperthermia may directly lead to cell death, with apoptosis believed to play a key role—mainly through the activation of procaspase-2 and a downstream cascade involving Bax or Bak proteins [7].

1,25(OH)_2_D_3_ (calcitriol), the active form of vitamin D_3_, regulates calcium metabolism and influences cell proliferation, differentiation, apoptosis, oncogenesis, and angiogenesis [8, 9]. The pleiotropic effects of 1,25(OH)_2_D_3_ are mediated by the vitamin D receptor (VDR), which, once activated, functions as a transcription factor regulating numerous cellular processes [10]. It is currently hypothesized that functional VDR expression is essential for the anti-tumor effects of 1,25(OH)_2_D_3_.[11]. VDR is expressed in most tumors, and numerous studies suggest that higher VDR expression correlates with better prognosis and longer overall survival, whereas reduced or absent VDR expression is associated with tumor progression and poorer outcomes [12].

The study employed human and murine pancreatic cancer cells, a human breast cancer cell line, and syngeneic Panc02 tumors to evaluate the effects of multifactorial therapy. Pancreatic cancer is a highly lethal disease and has a fatal prognosis [13]. The early diagnosis and treatment of this disease are extremely difficult because patients rarely show symptoms, and pancreatic cancers do not display sensitive and specific markers [13]. Current treatment strategies are unsatisfying, and new drugs are not a guarantee of success due to problems with, e.g., vascular invasion. Breast cancer is one of the most common diseases in women and is additionally one of the three most common cancers in the world [14]. It is also the most common cause of death among women and accounts for 23% of all deaths from cancer. Unfortunately, the morbidity and mortality due to this cancer have been increasing over the past 50 years [15].

The aim of this study was to ascertain the contribution of each therapeutic factor to the potency of multifactoral treatment in the context of breast and pancreatic cancer and to define the synergy effects of gemcitabine, hyperthermia, and 1,25(OH)_2_D_3_. Use of these three therapeutic approaches may open new translational possibilities, especially in the context of breast and pancreatic cancer and other cancers [16].

## Materials and methods

### Cell culture

Three cell lines were used in in vitro experiments: PANC-1 human pancreatic ductal adenocarcinoma (ATCC^®^), Panc02 murine pancreatic adenocarcinoma (DCTD Repository at Frederic National Laboratory for Cancer Research) and 4T1 mouse triple-negative breast cancer cells (ATCC^®^). Cells were cultured under standard conditions in humidified incubator at 37°C with 5% CO_2_. PANC-1 cells were cultured in DMEM high glucose media (Sigma-Aldrich, St. Louis, MO, USA), Panc02 and 4T1 cells were cultured in RPMI media (Sigma-Aldrich, St. Louis, MO, USA). Media were supplemented with 10% fetal bovine serum (Biological Industries, Cromwell, CT) and penicillin (100 unit/ml) and streptomycin (0.1 mg/ml) (Sigma-Aldrich, St. Louis, MO, USA).

### Treatment

The cells were seeded into 48-well (1×10^4^ Panc02 and 4T1 cells or 2×10^4^ PANC-1 cells per well) or 6-well plates and cultured in standard conditions for 24 hrs. Afterward, the cells were treated and cultured in standard conditions for up to 120 hrs. For hyperthermia, the cells were heated to 41°C for 30 minutes on a heating plate (SD2800 Slide Dryer, Histo-Line Laboratories, Italy). Next the cells were cultured at 37°C with 5% CO_2_ for 30 min. Cells were treated with gemcitabine (Sigma-Aldrich, St. Louis, MO, USA) at concentrations of 0.001 μM, 0.01 μM, 0.1 μM, 1 μM, 10 μM, 100 μM and 1000 μM and 1,25(OH)2D3 (Sigma-Aldrich, St. Louis, MO, USA) at 100 nM. The solutions of the drugs were prepared in the appropriate culture medium DMEM or RPMI with 10% fetal bovine serum.

### Cell Counting

At different time-points (24, 48, 72, 96 or 120 hrs) the cells were removed by incubation with 0.25% trypsin (Sigma-Aldrich, St. Louis, MO, USA) for one minute and then counted using the Bürker hemocytometer (Heinz Herenz, Hamburg, Germany). The experiment was performed in triplicate.

### Real-time Live Cell Imaging

The treated cells were imaged with an automatic light microscope as well as in the DAPI fluorescence channel for 6 days. For the imaging of cells in the DAPI channel (excitation parameters 390/40 nm and emission parameters 452/45 nm), Hoechst 33342 (Thermo Fisher Scientific, USA) was used. The cells were stained with Hoechst dye at a concentration of 50 μg/ml. 5 imaging positions in each well of the 48-well plate were selected. For each selected position in the well, a 3×3 matrix was imaged, which gave the final nine images from one position. Over 15% of the area was imaged through this solution from each well of the culture plate. A 10x magnification lens was used. Four independent replications of the experiment were performed.

### The MTT Assays

At each timepoint (24, 48, 72, 96 or 120 hrs) the cells were supplemented with 10% of MTT tetrazolium dye (3-(4,5-dimethylthiazol-2-yl)-2,5-diphenyltetrazolium bromide) (Sigma-Aldrich, St. Louis, MO, USA) stock solution (0.5 mg/ml) and incubated for 1.5 hours. The MTT formazan crystals that formed were dissolved in DMSO (Avantor, Poland) and methanol (Avantor, Poland) solution (1:1). Absorbance was measured at a wavelength of 565 nm with the Tecan Infinite 200Pro plate reader (Tecan, Switzerland). Four independent experiments were conducted.

### Western blot

The Western Blot was performed 48 hours after the cells had been treated. Cell monolayers were lysed on ice in lysing buffer that contained 1 mM of protease inhibitor cocktail (Roche, Switzerland), 0.1 mM PMSF (Sigma-Aldrich, St. Louis, MO, USA), 1 mM sodium orthovanadate (Sigma-Aldrich, St. Louis, MO, USA), and RIPA buffer (containing nonylphenol ethoxylates, Thermo Fisher Scientific). The cells with the lysis buffer had been incubated on ice for 15 min and then frozen at − 80°C for 5 min. The cells were then thawed on ice and centrifuged at 15,000 RPM for 10 min at 4°C. The amount of protein was measured using Bicinchoninic Acid (BCA) (Sigma-Aldrich, St. Louis, MO, USA) and Copper II sulfate (Sigma-Aldrich, St. Louis, MO, USA) and stored at −80°C until used.

Electrophoresis was performed on Mini Gel Tank (Thermo Fisher Scientific, USA). Proteins were run on Bolt^™^ Mini Protein Gel Bis-Tris (Thermo Fisher Scientific, USA). The gels used were gradient and contained from 4% to 12% polyacrylamide. The electrophoresis was carried out at a voltage of 200 V. A wet transfer was carried out, which lasted 1 hr and was performed at 10 V. For this purpose, nitrocellulose membranes were used. The proteins were blocked with 5% non-fat milk in a TBS-Tween 0.1% buffer for 1 hour. The next step was incubated with the primary antibodies at a dilution of 1:1000. Incubation with primary antibodies was overnight at temperature 4°C. Then, the membrane was washed three times in TBS-Tween 0.1% and incubated with the suitable secondary antibodies at a dilution of 1:2000 for 1.5 hrs.

The HRP (horseradis h peroxidase) detection system was used. The signals were detected chemiluminescence using a Super SignalTM West Pico PLUS Chemiluminescence Substrate (Thermo Fisher Scientific, Waltham, MA, USA). Membrane stripping was also performed. The stripping procedure was as follows. After signals are detected, the membranes were rinsed with distilled water for 5 minutes. The membranes were then washed in TBS-T for 10 minutes at room temperature. After this time, the membranes were washed in 0.5 M NaOH solution for 15 minutes. The membranes were then rinsed twice in TBS-T for 10 minutes. After this time, the membranes could be blocked again with 5% non-fat milk. Quantitative protein analysis was performed using ImageJ^®^ (Wayne Rasband, National Institute of Health, Bethesda, MD, USA).

### Flow cytometry

Panc02 line cells were seeded in 6-well plates (TPP, Switzerland) at a density of 10^5^/well in 2.5 ml of complete RPMI medium with fetal bovine serum. 24 h after seeding, cells were treated with therapy components. Gemcitabine doses were narrowed to 0.001 μM, 0.01 μM and 100 μM, while calcitriol was consistently used at 100 nM. The incubation time with the tested solutions was 48 h. To determine the type of cell death, the BD Pharmingen^™^ PE Annexin V Apoptosis Detection Kit I (Becton Dickinson, USA) was used. The kit was based on the use of PE Annexin V, which binds to apoptotic cells, and 7-AAD, which has an affinity for necrotic cells. Positive controls for apoptosis were cells treated for 6 h with a solution of Camptothecin in DMSO at a concentration of 6 μM. The experiment was performed according to the protocol provided by the manufacturer. Sample analysis was performed on a BD LSRFortessa^™^ flow cytometer (Becton Dickinson, USA) using BD FACSDiva^™^ 8.0 software.

### Animals and tumor inoculation

In vivo experiments were conducted using C57BL/6J mice, in which the tumor was orthotopically implanted in the pancreas. Males of approximately 8 weeks of age were used in the experiments. The animals were kept in SPF facility at a constant temperature (22 ± 2°C) and air humidity (55 ± 10%). The light day was 14 hours, and the darkness day was 10 hours. The animals had unlimited access to water and feed Altromin 1319 FORTI – Breeding Diet (ANIMALAB).

Panc02 cell spheroids were created by the suspended drop method in the amount of 3 × 10^3^ cells. They were cultured in standard culture conditions (37°C, 5% CO_2_) for 4 days. On the day of planned inoculation, spheroids were suspended in drops of solubilized basement membrane preparation, so-called Matrigel (Corning^®^ Matrigel^®^ Basement Membrane Matrix, Corning, USA) and then incubated for 45 minutes in an incubator (37°C, 5% CO_2_) to polymerize the matrix and form solidified fragments.

Before the operation, painkillers were administered (Buprenorphine, 0.6 mg/kg, subcutaneously in the neck area), (Bupivacaine, 1.0 mg/kg, subcutaneously in the area of the planned incision). The animals were anesthetized by inhalation of the anesthetic agent Isoflurane (0.5–5% in air) (Vetpharma Animal Health S.L., Spain). An approximately 5 mm skin incision was made at a safe distance under the spleen and an incision in the peritoneum at an angle of 60–90 degrees to the skin incision. Then, a fragment of Matrigel^®^ with an embedded spheroid of cancer cells was placed in the pancreas under the spleen. Dissolvable sutures (polyglycolide) were placed on the peritoneum, while non-dissolvable silk sutures and wound closure clips (AgnThos, Sweden) were used to close the skin.

All procedures were carried out based on the consent of the ethics committee no. 250/2020 and 353/2022 issued by the 2nd Local Ethical Committee for Animal Experiments in Krakow.

### Ultrasound imaging of tumor growth

Imaging was performed twice a week every 3–4 days from approximately day 15 of tumor cell inoculation until euthanasia of the animal. Animals were induced into anesthesia by steam inhalation via the anesthetic agent Isoflurane. Ultrasound imaging was performed using: Vevo2100 (FUJIFILM VisualSonic, Canada), Vevo F2 (FUJIFILM VisualSonic, Canada), Prospect T1 (Scintica, Canada), SmartUs EXT-1M (TELEMED, Lithuania). Different ultrasound devices were used due to their availability.

Correlation analysis between tumor mass and final ultrasound measurements confirmed that although average tumor diameter (X) varied within a relatively narrow range (1–10 mm), tumor volume (V) spanned over three orders of magnitude (1–1000 mm^3^), both parameters were strongly associated with actual tumor mass. These findings underscore the reliability of ultrasound in monitoring tumor burden and highlight the substantial therapeutic effect of the triple regimen not only in limiting tumor growth, but also in reducing disease spread.

### Therapy in vivo

Animals were classified for treatment when the ultrasound image showed the presence of a tumor with a volume ranging from about 5 mm^3^ to 30 mm^3^. Animals were assigned to specific therapeutic groups in a randomized manner, using the Statistica 13 TIBCO Software Inc. (StatSoft, Poland) program and the Experimental Planning and Analysis (DOE) function using the Complete Plan option.

The therapy consisted of 6 doses of the drug administered every 72 hours. Gemcitabine was administered at a concentration of 45 mg/kg of animal body weight into the tail vein. Local contact hyperthermia under inhalation anesthesia at a temperature of approximately 41°C for 30 minutes. Hyperthermia was performed using a heating lamp emitting infrared radiation. A 250 W bulb (Incandescent 230–250V BR125 Philips, Netherlands) approved for use in animals was used, as a heat radiator. The distance of the heat source from the animal was 30 cm. The temperature of the heated tissue with the tumor was controlled using a thermal imaging camera (FLIR). Calcitriol was administered to animals at a concentration of 100 nM as a solution in 0.9% physiological saline solution (Polpharma, Poland) in a dilution of 1:1000 in a volume of 2 ml intraperitoneally.

### Blood morphology

The tip of the tail was cut with a sharp, disposable scalpel and two drops of blood were collected from the tail vein. The tail was secured by applying pressure and/or Surgibond tissue glue (SMI, Germany). Blood samples were analyzed using the HoribaVet ABC Hematology Analyzer (HORIBA ABX, Japan). The following peripheral blood morphological parameters were measured: WBC, RBC, RDW, PLT), MPV, MON, MON%, MCV, MCHC, MCH, LYM, LYM%, HGB, HCT, GRA, GRA%.

### Euthanasia and tissue collection

Ten days after the end of therapy, the animals were euthanized pharmacologically by an overdose of a 1:1 ketamina:ksylazyna anesthetic mixture Bioketan 100 mg/ml, (Vetoquinol, Poland) and Sedazin 20 mg/ml (Biowet, Poland). After finding no response to pain and no consciousness, the cervical vertebrae were dislocated. Then, a dissection was performed, during which blood, tumor, pancreas, spleen, both kidneys, liver and heart were collected. Tumors and organs were weighed on a laboratory scale WTB 200, d=0.001 g (Radwag, Poland). The tissues were fixed in 10% formalin solution (POCH, Poland) for 72 hours and then transferred to 0.9% saline solution and stored at 4°C until further analysis.

### HE staining

The tissues were dehydrated and paraffinized. The paraffin blocks (Sigma-Aldrich, St. Louis, MO, USA) with tissues were cut into 6 μm slices and deparaffinized. Hematoxylin (Chempur, Poland) and Eosin (POCH, Poland) staining was performed using a standard procedure. Images of the specimens were taken using the Glissando^®^ Scanner (Objective Imaging Ltd, UK) using the Glissando Virtual Specimen Scanning^®^ software. Tissues were analyzed microscopically for structural features, in particular for the presence, size, and distribution of cells and the presence of tissue components.

### Synergy Effect Analysis

Data from in vitro and in vivo experiments were loaded into a Python programming environment. The following packages were used to load data, analyze and visualize results: pandas, numpy, matplotlib, seaborn and sklear. To determine whether the multifactorial therapies used had synergistic or additive effects, the Bliss independence model was used. Before using the above model, the data were normalized. According to the adopted Bliss model, when the predicted efficacy of combined therapies as independent factors was lower than the efficacy observed in the experiment (observed/predicted ratio > 1), the interaction was considered synergistic. If the observed efficacy did not differ from the predicted efficacy, the therapies were considered to have an additive effect (observed/predicted ratio = 1). When the efficacy of therapy predicted by Bliss’s model was higher than the efficacy observed during the experiment (observed/predicted ratio < 1), it was considered that the impact was antagonistic.

### Statistical analysis

In the first step, the distribution of the collected data types was checked using the Shapiro–Wilk or Jarque-Bera test, using STATISTICA, StatSoft (Timberlake, Poland). The analysis of statistical significance of the results was performed. The effects of all factors (including time and cell type) in the cell counting methods in the hemocytometer, MTT test, and real-time live cell imaging were studied by the multi-way ANOVA (script prepared by author ISD). The same data was also analyzed by estimating parameters of the generalized linear model (GEE, Generalized Estimated equation). GEE was developed specifically for analyzing longitudinal or clustered data where observations within the same group may be correlated, therefore it was particularly appropriate for our analysis as it accommodates repeated measurements over time. The statistical analysis of the VDR and HSP70 protein levels was performed using the ANOVA test (using STATISTICA). Statistical analysis of the type of cell death was performed using the One-Way ANOVA to compare means between cell death groups (Q1-Q4) and Tukey’s HSD test as post-hoc test and Kruskal-Wallis test as nonparametric alternative.

For the estimation of death risk of mice treated with multimodality treatment, Cox proportional hazards model was used (script prepared by author GD). The ratio of tumor volume at the time of imaging (V_t_) to tumor volume at classification (V_0_) was used to estimate therapy efficacy. Tumor volume 20 times that of the initial volume at the start of the therapy (V_t_/V_0_ ≥ 20) was considered therapy failure. The influence of the following factors on the risk of death was calculated: number of tumor foci, presence of ascites, age at the time of therapy initiation, tumor diameter at the time of classification into a given experimental group, initial mouse weight, standard deviation of weight during therapy, number of days from tumor cell inoculation to the start of therapy, and 8 different combinations of the treatment factors, including saline control. The Kruskal–Wallis’s test and a multiple comparison of mean ranks was used for the studied effect of tumor size: mid-therapy, at the end of therapy and around 10 later (before euthanasia). The analysis of differences in the number of tumor foci between groups of animals subjected to different therapies was also done using the Kruskal–Wallis’s test.

The analysis of blood morphology of mice subjected to therapy, collected at different time points, was performed. For data with normal distribution, the Student’s t-test was used (unpaired version for means from two independent groups or paired version for means from the same group subjected to different scenarios). In the case of non-normal distribution, the Wilcoxon rank-sum test (paired samples) or the Mann-Whitney test (unpaired samples) was used.

For all types of analysis, the significance level was p < 0.05.

## Results

### Hyperthermia and calcitriol induced HSP70 and VDR

**Fig. 2.**
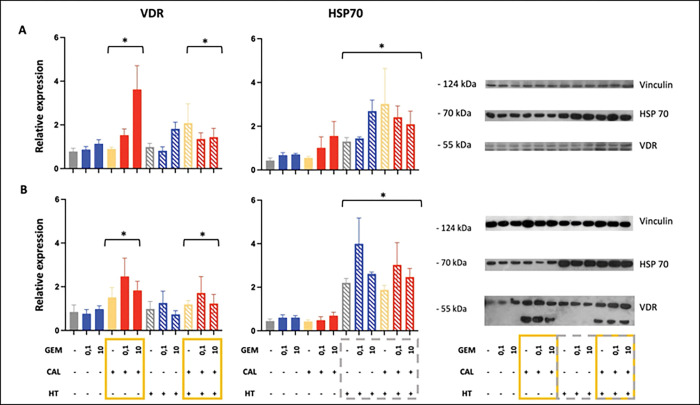
Molecular response to combined treatment. VDR (left) and HSP (right) protein levels in PANC-1 (A) and Panc02 (B) cells, determined from Western blot. Representative blot shown on the right. Vinculin was used as a reference protein. Gray – saline, blue – gemcitabine, yellow – calcitriol, red – gemcitabine and calcitriol, dashed filling indicates inclusion of hyperthermia. Statistical multi-way ANOVA: VDR – CAL *p* = 0,006 GEM *p* = 0,016; HSP70 – HT *p* = 0,001 (PANC-1); VDR – CAL *p* = 0,008; HSP70 – HT *p* = 0,000 (Panc02).

To evaluate the impact of treatment on protein-level responses, expression of the vitamin D receptor (VDR) and heat shock protein 70 (HSP70) was assessed by Western blotting, with densitometric quantification normalized to vinculin (Fig. 2). Calcitriol significantly upregulated VDR expression in all three tested cancer cell lines: Panc02 (p < 0.008), PANC-1 (p < 0.007), and 4T1 (p < 0.002). Gemcitabine alone increased VDR level only in PANC-1 cells (p < 0.016), whereas hyperthermia alone and the other two-factor combinations did not affect VDR levels in any of the models. Three-factor treatment of PANC-1 cells significantly increased VDR (p < 0.014).

As expected, HSP70 expression was modulated primarily by hyperthermia, with significant upregulation in both Panc02 and PANC-1 cell lines(Fig. 2). However, in 4T1 cells, the HSP70 response was more restricted: hyperthermia alone raised expression to 1.59, but neither dual nor triple treatments further enhanced it. Due to data variability and limited sample sizes in the 4T1 series, no definitive statistical conclusions could be drawn for this line.

Collectively, these findings show calcitriol as the dominant inducer of VDR, and hyperthermia as the primary activator of HSP70 in pancreatic tumor cells.

### Cellular Response to Triple Treatment

Morphological and quantitative analyses of cell cultures treated with the combination of calcitriol, hyperthermia, and gemcitabine demonstrated the most pronounced cytotoxic effects across all tested lines. In Panc02 and 4T1 cells, changes were visible as early as 24 hours post-treatment: cells appeared shrunken, membrane-damaged, or detached, often exhibiting complete loss of confluency (Fig. S1).

Notably, continuous monitoring of cell morphology and confluence showed that even the lowest gemcitabine concentration (1 nM) in triple treatment led to a substantial reduction in viable cell number, with no significant improvement observed at higher doses, indicating a plateau of maximal effect. At 72 hours, cells were either entirely absent or present as residual debris. In contrast, PANC-1 cells—known for slower proliferation—showed delayed morphological changes, with visible reduction in confluency only at 48–72 hours. After triple treatment with gemcitabine at 1 nM–1 μM, confluency dropped to 20% (Fig. S1). Real-time imaging (JuLI^™^ Stage) confirmed that neither calcitriol nor hyperthermia alone, nor their combination, significantly influenced cell proliferation over time. When combined with gemcitabine, a non-significant downward trend in Panc02 and 4T1 cell counts was observed at later time points and higher gemcitabine doses, while PANC-1 cell numbers remained unchanged or slightly elevated (Fig. S2).

**Fig. 3.**
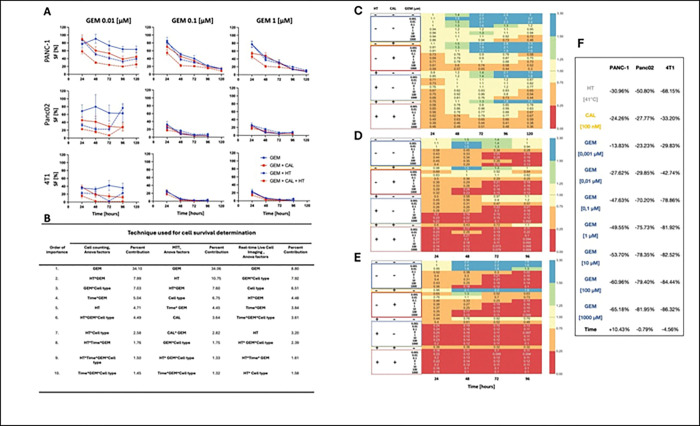
Quantitative analysis of triple therapy effects in cancer cell lines. (A) Surviving fraction of PANC-1, Panc02 and 4T1 cells determined by cell counting for selected concentrations of gemcitabine. Blue gemcitabine, dashed blue – gemcitabine and hyperthermia, red – gemcitabine and calcitriol, dashed red-gemcitabine, calcitriol and hyperthermia. Only selected experimental groups are shown. (B) Parameters obtained from 5-way ANOVA for the ten most important effects (P value<0.05) influencing the cell survival determined either by cell counting, MTT or real-time live cell imaging. Results from all experimental groups are included. Effects are listed in a descending order of the percentage of explained variance. Relative metabolic activity of PANC-1 (C) Panc02 (D) 4T1 (E) treated with gemcitabine (0,001 μM – 1000 μM), calcitriol (100 nM), hyperthermia (41°C). (F) GEE regression coefficients (%) showing treatment effects on cell viability across three cancer cell lines.

Quantification of cell number confirmed these observations (Fig. 3A). In Panc02 cells, survival dropped to 13.44 ± 2.25% after 24 hours at 1 nM gemcitabine, and further declined with higher doses (e.g., 7.75 ± 0.83% at 1 mM). Similarly, PANC-1 cells responded with significant survival reduction from 40.64 ± 2.35% (1 μM, 24 h) to as low as 5.28 ± 1.33% at 120 h (10 μM). For 4T1 cells, the response was most rapid and potent: survival fell below 2% by 72 hours across all gemcitabine concentrations. These data emphasize that 4T1 cells are the most sensitive, with effects evident even at nanomolar concentrations.

Assessment of the metabolically active cell fraction corroborated viability trends. In Panc02 cells, metabolic activity declined to 11.54 ± 0.63% – 18.85 ± 0.86% at 24 hours, with progressive reduction at 48, 72 and 96 h. Similar trends were observed in PANC-1 cells, with a delayed response. In 4T1 cells, metabolic activity was consistently suppressed to below 20% across all time points and concentrations. These patterns confirm robust enhancement of gemcitabine efficacy by calcitriol and hyperthermia, particularly in rapidly dividing tumor cells (Fig. 3C– E).

In Panc02 cells, hyperthermia and calcitriol induced early (10.43 ± 1.22%) and late (39.90 ± 7.04%) apoptosis, with minimal necrosis (1.63 ± 0.23%). Hyperthermia with low-dose gemcitabine also increased apoptosis. Triple treatment with 1 nM gemcitabine slightly raised late apoptosis (36.57 ± 4.72%), while 100 μM gemcitabine further elevated both late apoptosis (48.98 ± 3.75%) and necrosis (2.30 ± 0.72%). Similar patterns were observed in 4T1 cells, where triple therapy with 100 μM gemcitabine resulted in 66.43 ± 0.90% late apoptotic and 3.75 ± 0.72% necrotic cells. ANOVA revealed significant differences (p < 0.05) across groups for early apoptosis, late apoptosis, and viable cells in both Panc02 and 4T1 lines, with the most significant differences (p < 0.001) observed between high-dose treatments and controls. Combined therapies, particularly triple treatment, differed significantly from monotherapies. In 4T1 cells, necrosis showed fewer significant differences, though still statistically meaningful (ANOVA p = 0.0089). For Panc02 cells, necrosis was not significant (ANOVA and Kruskal-Wallis p > 0.05). These results highlight the synergistic pro-apoptotic effect of 1,25(OH)_2_D_3_ and hyperthermia, especially when combined with gemcitabine (Fig. S3).

In summary, triple therapy (gemcitabine, 1,25(OH)_2_D_3_, hyperthermia) elicited potent cytotoxic effects through induction of apoptosis, reduction in metabolic activity, and inhibition of cell proliferation. The 4T1 model proved most responsive, while PANC-1 showed delayed but sustained effects, highlighting differences in therapy sensitivity related to proliferative capacity and origin of cancer cells.

### Contribution of the three factors to overall cytotoxicity

The 5-way ANOVA analysis conducted using our *in vitro* data revealed that among all tested conditions, the concentration of gemcitabine was the most critical factor determining cancer cell survival (percent of contribution for cell counting and MTT was 34%, Fig. 3B). This effect was consistently strong across all three experimental assays—manual cell counting in a hemocytometer (34%), assessment of metabolic activity via MTT assay (34%), and nuclear staining for cell quantification (8,8%). In each method, gemcitabine alone accounted for the largest share of variability in cell viability outcomes, emphasizing its central role in the treatment response. The timing of measurements (i.e., the duration of gemcitabine incubation) also emerged as a statistically significant contributor, particularly in assays where delayed drug effects were expected (between 3.8–5% of contribution). Cell type contributed to MTT and real-time live cell imaging at a level of 6.5%. Independent of the method of cell survival measurement, hyperthermia is a relevant factor and contributes to anticancer effects; however, the percentage of contribution varies between 10.75% and 3.2%. 1,25(OH)_2_D_3_ contributes only to the decrease of metabolic activity by 3.64%. While 1,25(OH)_2_D_3_ and hyperthermia alone had less pronounced effects, their influence became more important when combined with gemcitabine, indicating that their role lies primarily in modulating or enhancing chemotherapy efficacy. Notably, interaction effects—especially between gemcitabine and hyperthermia or 1,25(OH)_2_D_3_ —showed statistically significant contributions to overall treatment outcomes, reinforcing the idea that combination therapies can achieve greater efficacy than single-agent treatments alone. Overall, this statistical analysis highlights that gemcitabine dose is the primary driver of cell death, but combinatory strategies significantly improve the therapeutic profile (Fig. 3).

**Fig. 4.**
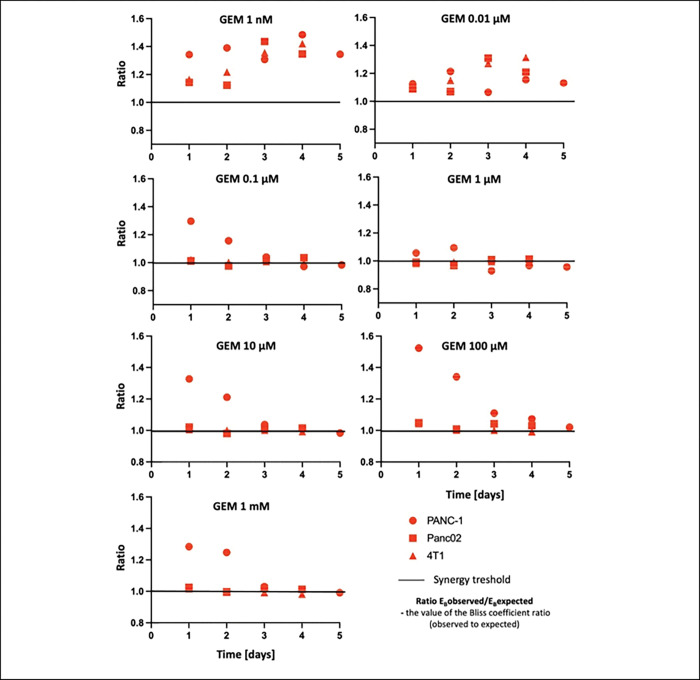
Bliss synergy analysis. Results of Bliss effect analysis for three-factor treatment of PANC-1 (circles), Panc02 (squares), and 4T1 (triangles) cells presented for the range of gemcitabine dose. A value of 1.0 on the Y scale indicates the synergy threshold. Points above the synergy threshold indicate a synergistic effect, those on the line correspond to an additive effect, and those below the line indicate antagonistic effects. Results obtained based on the MTT test.

To account for the temporal aspect in therapy, we conducted a statistical analysis using the Generalized Estimating Equations (GEE) method with Poisson family distribution. All reported treatment effects demonstrated statistical significance (p < 0.001). Detailed data are presented in Fig. 4, while the complete GEE analysis results can be found in Fig. S4 and S5.

Our GEE analysis revealed significant differences in treatment response profiles among the three examined cancer cell lines. Temperature elevation to 41°C substantially affected all cell lines, with the strongest impact observed in 4T1 (−68.15%), followed by Panc02 (−50.80%), while Panc01 showed comparatively lower sensitivity (−30.96%). Calcitriol treatment showed variable effects across cell lines, with inhibition percentages ranging from 24.26% in Panc01 to 33.20% in 4T1.

The main GEE contribution to cytotoxicity was exerted by gemcitabine, consistent with the ANOVA results. The gemcitabine concentration-response relationship exhibited notable differences between cell lines. 4T1 demonstrated the highest sensitivity to gemcitabine, with maximum inhibition of 86.32% at 1000 μM, followed by Panc02 (81.95%), while Panc01 was relatively less sensitive (65.18%). Interestingly, all three cell lines exhibited a plateau effect in response to gemcitabine concentrations above 0.1–1 μM, suggesting that moderate rather than maximum doses might be sufficient for optimal therapeutic efficacy.

The temporal dimension of treatment response also differed significantly between cell lines. While Panc01 showed a significant positive day coefficient (10.43%, p < 0.001), reflecting different growth kinetics, both 4T1 and Panc02 showed negative time-dependent changes in viability (−4.56%, p = 0.677 and − 0.79%, p = 0.943, respectively). These findings highlight the importance of cell-specific treatment strategies and suggest that combination therapy incorporating hyperthermia with moderate gemcitabine concentrations may represent an effective approach for targeting multiple cancer types, particularly those resistant to single-agent treatments.

Analysis of the Bliss effect for the triple treatment shows a synergistic effect on metabolic activity (Fig. 4) and cell counting (not shown). The synergy is strongest for low doses of gemcitabine (1 nM and 10 nM). Importantly, only resistant human cells, PANC-1, exhibited a synergistic effect for the whole range of gemcitabine concentrations, which can be related to a weak response to the drug given alone or in two-factorial treatment. For all cell lines at low GEM doses, the synergy is seen for at all time-points (up to 5 days), whereas for PANC-1 cell it is the strongest for the first 48 hours.

### Inhibition of PDAC tumor growth and enhancement of animal survival

**Fig. 5.**
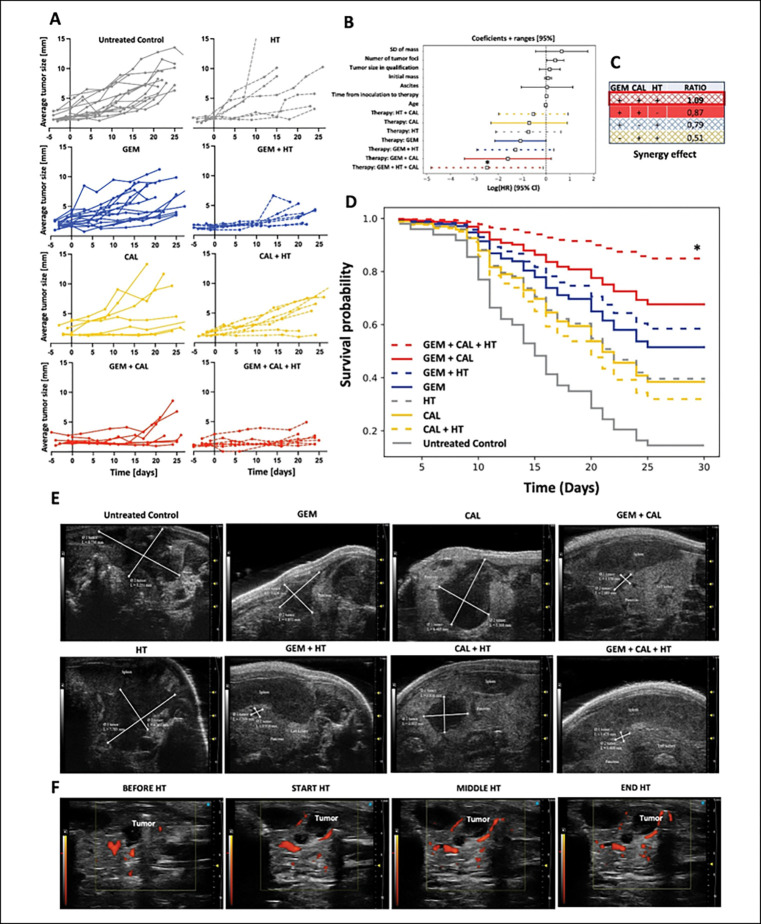
Animal survival and tumor imaging during anticancer therapy. (A) Growth kinetics of tumors in mice during and after therapy. Day 0 is the time of therapy initiation. Tumor size [mm] was measured by ultrasonography. (B) The effect size for each factor studied was assessed as the relative risk (log HR) of death of an animal with tumor. Data were obtained based on the likelihood ratio test. (C) Analysis of the effect of interactions using the Bliss Independence Model based on tumor volume (V/V0) before euthanasia. (D) Probability of survival of tumor-bearing animals by experimental group based on Cox analysis. Day 0 represents the time of therapy initiation. (D) (E) Ultrasound images of animals with tumors located in the pancreas before euthanasia. The size of tumors was marked by marking the diameters at the widest point of the tumors with the ultrasound probe positioned parallel (cross-section). (F) Hyperthermia-induced flow changes using Doppler ultrasound (Power Doppler). The intensity and area of the red signal are directly related to the recorded flow in the tissue area marked with a square.

In the control group receiving saline, tumors grew steadily but with high variation between animals, reaching an average diameter of 8.45 ± 0.53 mm by the end of the experiment. In contrast, gemcitabine monotherapy (45 mg/kg BW) moderately reduced tumor size, with tumors increasing from 3.53 ± 0.45 mm at mid-therapy to 6.82 ± 0.67 mm before euthanasia. Although tumor growth was not fully suppressed, survival improved, with approximately 50% of treated animals alive 30 days after treatment initiation. Cox regression analysis showed a hazard ratio coefficient of −1.07 ± 0.55, indicating a modest survival benefit from gemcitabine alone (Fig. 5).

1,25(OH)_2_D_3_ alone at 100 nM had no impact on tumor progression, with final tumor size closely resembling the control group. However, the combination of 1,25(OH)_2_D_3_ with gemcitabine led to a marked reduction in tumor burden, as evidenced by a final tumor mean diameter of 4.22 ± 1.07 mm. This dual-treatment group demonstrated a survival probability of approximately 70%. Corresponding hazard ratio coefficients were not significant.

Hyperthermia applied as a single modality exhibited no therapeutic benefit. In contrast, its combination with gemcitabine substantially reduced tumor size (3.84 ± 0.55 mm) and improved survival to approximately 60%. When paired with 1,25(OH)_2_D_3_, hyperthermia had no effect (5.75 ± 1.17 mm, V/V₀ = 112.3), with an associated survival of ~ 30%. Mechanistically, Doppler ultrasound imaging confirmed increased tumor perfusion and vessel dilation following hyperthermia (Fig. 5F), supporting the role of heat as a physiological modulator enhancing drug delivery.

Triple-modality therapy, combining gemcitabine, 1,25(OH)_2_D_3_, and hyperthermia, achieved the most pronounced anti-tumor effects. At the study endpoint, tumors were significantly smaller (2.44 ± 0.48 mm; V/V₀ = 6.44), and animal survival increased to approximately 85%. Cox regression analysis yielded the most favorable effect size (coef = −2.47 ± 1.20; p = 0.0398), confirming the synergistic efficacy of the triple regimen in both tumor suppression and increased survival.

Further analysis of individual variables revealed that an increased number of tumor foci (coef = 0.39 ± 0.18), body weight loss (coef = 0.66 ± 0.56), and the presence of ascites (coef = 0.05 ± 0.55) were not associated with higher mortality risk. Similarly, baseline tumor diameter, initial body weight, time from tumor inoculation to treatment, and animal age had negligible impact on survival outcomes (all coef values approximating zero; Fig. 5B).

### Beneficial response to therapy is linked to control over lymphocytes

**Fig. 6.**
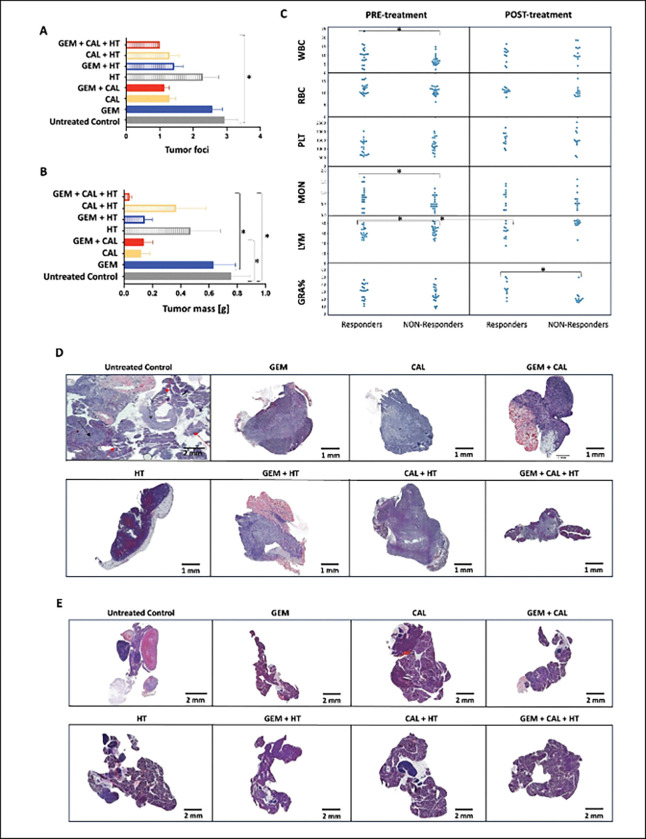
Ex vivo assessment of tumor burden, systemic effects, and histopathology in treated animals. (A) Number of tumor foci in animals in the pancreas and in adjacent organs in the post-mortem examination. Marked with * p-value<0.05 for the Kruskal-Wallis statistical test. (B) Mass of tumor in the post-mortem examination. Marked with * p-value<0.05 for the Kruskal-Wallis statistical test. (C) Blood count parameters before and after therapy. (D) Example images of tumor and (E) pancreas after H&E staining. Slide images were taken using the Glissando^®^ Scanner using the Glissando Virtual Specimen Scanning^®^ software.

The impact of therapy on blood morphology was assessed by comparing pre- and post-treatment hematological profiles in mice, categorized as responders (tumor volume ratio V/V₀ < 20) or non-responders (Fig. 6C). Prior to treatment, responders exhibited higher red blood cell (RBC) counts and lower platelet (PLT) levels compared to non-responders. Following therapy, responders showed an increase in granulocyte levels. In contrast, non-responders had lower baseline levels of white blood cells (WBC) and monocytes. Post-treatment, these animals displayed elevated lymphocyte percentages and a reduction in granulocytes. These findings suggest that therapeutic efficacy may be associated with a coordinated hematological shift, particularly involving the regulation of lymphocyte and granulocyte populations.

### Triple therapy limits the number of tumor foci in the peritoneal cavity

Quantitative post-mortem analysis showed that the triple therapy significantly reduced the number of tumor foci per animal to a mean of one (p = 0.017), compared to approximately three in the untreated group (Fig. 6). Triple therapy treated tumors were significantly smaller in comparison to untreated control (p = 0.0001) and gemcitabine treated tumors (p = 0,0033). Interestingly, also mice treated with gemcitabine and 1,25(OH)_2_D_3_ presented significantly smaller tumors than untreated controls (p = 0,02821).

Tumors from control and hyperthermia-only groups frequently invaded surrounding tissues or displayed a diffuse morphology, as evidenced by extensive pancreatic infiltration seen in representative samples (Fig. 6). Histological staining revealed therapy-induced necrosis (regions of nuclear blurring). Importantly, in animals receiving the combined treatment, tumors were small and limited to the pancreas, in contrast to untreated controls, where cancer infiltration dominated the tissue.

## Discussion

The present study provides comprehensive evidence supporting the enhanced efficacy of combinatory therapy involving gemcitabine, hyperthermia, and 1,25(OH)_2_D_3_ in both in vitro and in vivo models of cancer. Our experiments demonstrated the efficacy of the triple combinatory treatment across different cellular contexts, with particular focus on overcoming chemoresistance in pancreatic ductal adenocarcinoma. The three factors are already present in the clinic, and therefore this approach is easy to implement in future patient treatment.

### Models and survival tests to study complementary treatment effects

To ensure biological relevance and experimental validity, we first selected cell lines with sufficient expression of the VDR, as confirmed by Western blotting. This ensured a rational basis for including calcitriol as a therapeutic modulator. Another factor taken into account was the varied doubling time of cells, as gemcitabine efficacy strongly depends on cell division. We employed three distinct cell lines to model variable treatment responses: Panc02 (murine PDAC, allowing direct in vivo translation), PANC-1 (human PDAC, highly resistant), and 4T1 (murine breast cancer, highly sensitive). Panc02 cells served as a translational bridge between murine and human systems, while the inclusion of 4T1 cells allowed us to assess the broad applicability of the treatment beyond pancreatic cancer.

Cell viability and metabolic activity were assessed using cell counting, MTT assays, and continuous real-time live cell imaging. Although these methods differ in their biological targets (cell number vs. mitochondrial activity), they consistently demonstrated a trend of enhanced therapeutic efficacy when all three treatment components were applied (Fig. 3, and Fig. S2). Despite some slight discrepancies between methods (especially in the real-time live cell imaging modality vs cell counting, where only live cells were included), the convergence of data supported the overall conclusions. Cytometry further validated apoptosis induction across treatments, particularly in 4T1 and Panc02 cells, aligning with known mechanisms of gemcitabine-induced cell death (Fig. S3) [17].

### Hyperthermia and calcitriol modulate gemcitabine cytotoxicity

Our findings confirmed the cytotoxicity of gemcitabine at concentrations of ≥0.1 μM with saturation of the response at higher doses. While 4T1 cells exhibited rapid and robust responses, PANC-1 cells required extended exposure and higher concentrations, reflecting their known chemoresistance [18]. Panc02 cells showed intermediate sensitivity, with durable effects observed after 48 hours. Importantly, low gemcitabine concentrations were more amenable to modulation by adjunct therapies, supporting their use in combinatory strategies.

Hyperthermia alone induced modest reductions in cell viability and a transient increase in HSP70 expression, confirming its biological impact. While its cytotoxic effect was limited, hyperthermia significantly enhanced the efficacy of gemcitabine in vitro and increased vascular perfusion in vivo, likely improving drug delivery. Our Western blot data confirmed HSP70 upregulation following hyperthermia, consistent with cellular stress responses [19].

1,25(OH)_2_D_3_ alone at fixed 100 nM concentration did not affect viability or metabolism but significantly increased VDR expression, providing mechanistic evidence of biological activity. Although ineffective as monotherapy, calcitriol enhanced gemcitabine efficacy in dual and triple treatments, particularly in the resistant PANC-1 line. This is in line with reports demonstrating 1,25(OH)_2_D_3_-mediated suppression of efflux transporters such as MRP1 and MRP5, leading to improved intracellular retention of chemotherapeutic agents [20]. The combination of calcitriol with gemcitabine has a synergistic effect on the proliferation of Capan-1 cells, which means that the combination strongly induced apoptosis: the activation of caspases-8, -9, -6 and -3 was significantly higher than in monotherapy, and at the same time, a significant decrease in Akt phosphorylation was observed, which weakens the signals of cell survival [21]. The rationale behind using a relatively high 1,25(OH)_2_D_3_ concentration (100 nM) in the study was based on using a single dose, as well as its fast degradation in the solution, and obtaining biological efficiency possible only with a high concentration [21–23]. Further in vivo studies might utilize non-calcemic derivatives, enabling high concentration in vivo without known calcemic effects of 1,25(OH)_2_D_3_ [24–29].

Our findings, supported by previous studies, suggest that gemcitabine may stimulate VDR expression. Overnight incubation with gemcitabine has been shown to upregulate VDR in PANC-1 pancreatic cancer cells [30]. However, cellular responses under hyperthermic conditions may differ due to stress-induced signaling. Notably, VDR was reported to interact with HSP90 in human leukemia cells, indicating a potential role for molecular chaperones in VDR regulation under stress [31]. Additionally, earlier studies demonstrated that VDR heterologously expressed in yeast binds to the cytosolic heat shock protein HSP70, specifically the Ssa1p isoform [32]. These findings suggest that heat shock proteins, such as HSP70 and HSP90, may influence VDR stability, localization, or function.

### In Vivo Therapeutic Impact and Mechanistic Insights

In vivo, triple therapy achieved the most effective tumor control. Panc02 tumors not only ceased growing or regressed but also showed a markedly reduced frequency of peritoneal metastases. Histopathological analysis confirmed the absence of organ infiltration—including the liver, kidneys, intestines, and peritoneum—in the triple therapy group, with residual tumor confined to the pancreas. In contrast, dual treatment with hyperthermia and calcitriol, excluding gemcitabine, did not produce comparable effects, highlighting the critical role of gemcitabine as the cytotoxic backbone of the therapeutic strategy [33].

Cox survival analyses demonstrated a stepwise increase in survival probability with increasing complexity of therapy. While gemcitabine monotherapy achieved ~ 50% survival, significant extension of survival was only seen with the triple regimen. Importantly, the selected gemcitabine dose allowed for in vivo efficacy without excessive toxicity, and adjunctive use of hyperthermia and 1,25(OH)_2_D_3_ enhanced this effect while minimizing side effects. High-dose chemotherapy or ablative hyperthermia can increase efficacy but come with toxicity risks, including immunogenic tissue damage [34] or 1,25(OH)_2_D_3_-induced hypercalcemia [35].

The proposed mechanisms include increased intratumoral drug accumulation through 1,25(OH)_2_D_3_-mediated effects [20], improved vascular perfusion from hyperthermia, and reduced drug clearance [36–38]. Furthermore, hyperthermia may suppress DNA repair via denaturation of MRE11, a key homologous recombination repair protein [39], and enhance gemcitabine activation by dCK through inhibition of EFNA4/β-catenin signaling [40]. Hyperthermia also induces G2/M arrest and apoptosis through intrinsic and extrinsic pathways [41], while 1,25(OH)_2_D_3_ modulates proliferation and apoptosis. Moreover, hyperthermia may activate deoxycytidine kinase (dCK), augmenting gemcitabine bioactivation [42].

Cox regression modeling identified that animals with fewer peritoneal lesions had higher survival, reinforcing the anti-metastatic efficacy of triple therapy. Body weight remained stable across groups, suggesting limited systemic toxicity. Ascites presence and hematological parameters also correlated with survival and could serve as predictive markers. Blood cell indices, possibly affected by splenic function, warrant further investigation, especially given the immune system and the role of anemia in tumor chemoresistance [43].

### Synergistic effects of triple treatment

The most significant findings emerged from the triple factor arm, where combinatory treatment with gemcitabine, hyperthermia, and 1,25(OH)_2_D_3_ elicited the strongest and most durable anti-cancer effects across all tested models. Statistical analyses revealed significant effects in cell count and metabolic assays from 24 to 120 hours post-treatment, particularly in the resistant PANC-1 cells. These data were corroborated by changes in morphology and confluency, as well as by apoptotic profiles.

Analysis using the Bliss independence model confirmed synergy at low gemcitabine concentrations, especially in PANC-1 cells. In contrast, mouse cell lines showed saturated responses even under monotherapy, limiting the detection of synergy. Notably, our in vivo data supported these in vitro findings: triple therapy led to tumor regression, reduced peritoneal dissemination, and increased survival without evidence of systemic toxicity.

Although Bliss synergy scores in vivo indicated only mild synergy (Bliss ratio ≈ 1.1) [44], this likely reflects the limitation of the model, which was based solely on tumor volume increase over time. Indeed, mono- and dual-agent treatments with hyperthermia sometimes led to paradoxical increases in tumor volume, which were effectively controlled in the triple combination, suggesting antagonistic effects were overcome. In our study, hyperthermia exhibited distinct effects in vitro and in vivo. In vitro, hyperthermic treatment primarily activated the apoptotic pathway, indicating a direct cytotoxic effect on tumor cells. In contrast, the predominant in vivo response to hyperthermia was an increase in perfusion within the heated tissue region. Notably, the distribution of heat in vivo was not uniform across the entire tumor volume, which may have led to heterogeneous thermal exposure [45, 46]. As a result, certain tumor regions may have experienced enhanced delivery of oxygen and nutrients due to improved vascular perfusion, despite limited or absent drug penetration in those areas. This discrepancy between thermal distribution and drug availability could influence therapeutic efficacy and should be considered when interpreting in vivo outcomes of hyperthermia-based treatments.

These multifactorial interactions highlight the potential of combinatory approaches to overcome drug resistance and improve therapeutic outcomes.

### Translational Relevance and Future Directions

The evidence presented underscores the therapeutic potential of triple therapy in resistant cancers such as PDAC. The observed synergy allows for effective tumor control at lower gemcitabine doses, reducing the risk of systemic toxicity. Moreover, the combinatory approach using sublethal doses of hyperthermia and 1,25(OH)_2_D_3_ circumvents the need for hyperthermia-induced ablation or calcemic calcitriol dosing, both of which are clinically challenging.

Further research is warranted to optimize dosing regimens and elucidate long-term outcomes, particularly elucidating the mechanism of action in immunocompetent and orthotopic tumor models. Future studies should also consider next-generation calcitriol analogs with reduced calcemic activity [47] or non-calcemic vitamin D_3_ hydroxyderivatives [9] produced not only in humans [48] but also across different species [49], and explore alternative synergy quantification models (e.g., Loewe, ZIP) to refine mechanistic insights [50].

## Conclusion

Our findings demonstrate that multifactorial therapy combining gemcitabine, hyperthermia, and calcitriol provides superior anti-cancer efficacy compared to monotherapies or dual combinations. While calcitriol alone had negligible cytotoxic impact, it enhanced the effectiveness of gemcitabine, particularly when hyperthermia was also applied. In vitro, this triple regimen was most effective at low gemcitabine concentrations shortly after thermal exposure, while in vivo it significantly reduced tumor growth, limited metastatic spread, and improved survival. The biological effects observed highlight the importance of temporal dynamics in multi-agent treatment response, the relevance of drug modulation at low-dose ranges, and the balance between the factors. Overall, our data confirm the additive and synergistic effects of combining gemcitabine with 1,25(OH)_2_D_3_ and hyperthermia and suggest this approach as a rational and effective strategy for enhancing treatment outcomes in aggressive and chemoresistant malignancies.

## Supplementary Material

Supplementary Files

This is a list of supplementary files associated with this preprint. Click to download.

• 4T1VDR2.jpg

• 4T1eks1PANC1eks124.jpg

• Supplement.docx

• PANC1VDR2.jpg

• 4T1HSP70.jpg

• PANO2VDR.jpg

• PANO2HSP70.jpg

## Data Availability

The dataset supporting the conclusions of this article is available in the RODBUK repository, https://doi.org/10.57903/UJ/S18L27

## Glossary

Apoptosis: A form of programmed cell death characterized by specific morphological and biochemical features, including cell shrinkage, membrane blebbing, and DNA fragmentation
Calcitriol (CAL): 1,25(OH)_2_D_3_ – the hormonally active form of vitamin D_3_, which regulates calcium metabolism and exhibits anti-proliferative and pro-differentiation effects in cancer cells through activation of the vitamin D receptor (VDR)
Caspases: A family of protease enzymes that play essential roles in apoptosis, with caspase-3 being one of the key executioners of this process.
Combination therapy: A treatment strategy involving the simultaneous use of two or more therapeutic agents or modalities to enhance efficacy and reduce resistance
Flow cytometry: A technique used to detect and measure physical and chemical characteristics of a population of cells or particles, commonly used to assess cell death and immune markers.
Gemcitabine (GEM): A nucleoside analog chemotherapeutic agent (dFdC) that inhibits DNA synthesis and induces apoptosis in rapidly dividing cells, often used in the treatment of pancreatic and breast cancers
Hyperthermia (HT): A cancer treatment modality that involves elevating the temperature of tumor tissues
HSP70 (Heat Shock Protein 70): A molecular chaperone upregulated in response to stress, including heat, which helps protect cells and is involved in protein folding and apoptosis regulation
MTT assay: A colorimetric assay for assessing cell metabolic activity as a proxy for cell viability, based on reduction of MTT dye to formazan by active mitochondria.
Normothermia (NT): standard temperature of cell culture or animal living (in this work, experimental groups in which hyperthermia was not used
PANC-1, Panc02, 4T1: Cancer cell lines used in the study: PANC-1 (human pancreatic ductal adenocarcinoma), Panc02 (murine pancreatic adenocarcinoma), and 4T1 (murine triple-negative breast cancer)
Survival Fraction (SF): percentage of cells remaining after exposure to therapeutic agents in comparison to untreated cells [%]
Synergy (Bliss model): A statistical interaction indicating that the effect of a combination of agents exceeds the expected additive effect, as evaluated by the Bliss independence model.
VDR (Vitamin D Receptor): A nuclear receptor activated by calcitriol, which modulates gene expression and contributes to the anti-tumor effects of vitamin D derivatives

## References

[1] Mokhtari R.B., Homayouni T.S., Baluch N., Morgatskaya E., Kumar S., Das B., Yeger H., Combination therapy in combating cancer, Oncotarget 8 (2017) 38022–38043. 10.18632/oncotarget.16723. 28410237 PMC5514969

[2] Blagosklonny M. V., Overcoming limitations of natural anticancer drugs by combining with artificial agents, Trends Pharmacol Sci 26 (2005) 77–81. 10.1016/j.tips.2004.12.002. 15681024

[3] Slominski R.M., Raman C., Chen J.Y., Slominski A.T., How cancer hijacks the body’s homeostasis through the neuroendocrine system., Trends Neurosci 46 (2023) 263–275. 10.1016/j.tins.2023.01.003. 36803800 PMC10038913

[4] Chandler N.M., Canete J.J., Callery M.P., Caspase-3 drives apoptosis in pancreatic cancer cells after treatment with gemcitabine., J Gastrointest Surg 8 (2004) 1072–8. 10.1016/j.gassur.2004.09.054. 15585396

[5] Shi X., Liu S., Kleeff J., Friess H., Büchler M.W., Acquired Resistance of Pancreatic Cancer Cells towards 5-Fluorouracil and Gemcitabine Is Associated with Altered Expression of Apoptosis-Regulating Genes, Oncology 62 (2002) 354–362. 10.1159/000065068. 12138244

[6] Cheng Y., Weng S., Yu L., Zhu N., Yang M., Yuan Y., The Role of Hyperthermia in the Multidisciplinary Treatment of Malignant Tumors, Integr Cancer Ther 18 (2019) 153473541987634. 10.1177/1534735419876345. PMC7242805 31522574

[7] Hildebrandt B., The cellular and molecular basis of hyperthermia, Crit Rev Oncol Hematol 43 (2002) 33–56. 10.1016/S1040-8428(01)00179-2. 12098606

[8] Holick M.F., Slominski A.T., Photobiology of vitamin D, in: Feldman and Pike’ s Vitamin D, Elsevier, 2024: pp. 27–45. 10.1016/B978-0-323-91386-7.00006-4.

[9] Slominski A.T., Kim T.-K., Janjetovic Z., Slominski R.M., Li W., Jetten A.M., Indra A.K., Mason R.S., Tuckey R.C., Biological Effects of CYP11A1-Derived Vitamin D and Lumisterol Metabolites in the Skin, Journal of Investigative Dermatology 144 (2024) 2145–2161. 10.1016/j.jid.2024.04.022. 39001720 PMC11416330

[10] Pike J.W., Meyer M.B., The Vitamin D Receptor: New Paradigms for the Regulation of Gene Expression by 1,25-Dihydroxyvitamin D3, Endocrinol Metab Clin North Am 39 (2010) 255–269. 10.1016/j.ecl.2010.02.007. 20511050 PMC2879406

[11] Slominski R.M., Kim T.-K., Janjetovic Z., Brożyna A.A., Podgorska E., Dixon K.M., Mason R.S., Tuckey R.C., Sharma R., Crossman D.K., Elmets C., Raman C., Jetten A.M., Indra A.K., Slominski A.T., Malignant Melanoma: An Overview, New Perspectives, and Vitamin D Signaling, Cancers (Basel) 16 (2024) 2262. 10.3390/cancers16122262. 38927967 PMC11201527

[12] BROŻYNA A.A., HOFFMAN R.M., SLOMINSKI A.T., Relevance of Vitamin D in Melanoma Development, Progression and Therapy, Anticancer Res 40 (2020) 473–489. 10.21873/anticanres.13976. 31892603 PMC6948187

[13] Kleeff J., Korc M., Apte M., La Vecchia C., Johnson C.D., Biankin A. V., Neale R.E., Tempero M., Tuveson D.A., Hruban R.H., Neoptolemos J.P., Pancreatic cancer, Nat Rev Dis Primers 2 (2016) 16022. 10.1038/nrdp.2016.22. 27158978

[14] Harbeck N., Gnant M., Breast cancer, The Lancet 389 (2017) 1134–1150. 10.1016/S0140-6736(16)31891-8. 27865536

[15] Donepudi M.S., Kondapalli K., Amos S.J., Venkanteshan P., Breast cancer statistics and markers, J Cancer Res Ther 10 (2014) 506–511. 10.4103/0973-1482.137927. 25313729

[16] Piotrowska A., Wierzbicka J., Rybarczyk A., Tuckey R., Slominski A., Żmijewski M., Vitamin D and its low calcemic analogs modulate the anticancer properties of cisplatin and dacarbazine in the human melanoma A375 cell line, Int J Oncol (2019). 10.3892/ijo.2019.4725. PMC6411347 30968156

[17] Mini E., Nobili S., Caciagli B., Landini I., Mazzei T., Cellular pharmacology of gemcitabine, Annals of Oncology 17 (2006) v7–v12. 10.1093/annonc/mdj941. 16807468

[18] Petrov M.S., Diabetes of the exocrine pancreas: American Diabetes Association-compliant lexicon, Pancreatology 17 (2017) 523–526. 10.1016/j.pan.2017.06.007. 28655595

[19] Dudeja V., Mujumdar N., Phillips P., Chugh R., Borja-Cacho D., Dawra R.K., Vickers S.M., Saluja A.K., Heat shock protein 70 inhibits apoptosis in cancer cells through simultaneous and independent mechanisms., Gastroenterology 136 (2009) 1772–82. 10.1053/j.gastro.2009.01.070. 19208367 PMC2896387

[20] Gilzad-Kohan H., Sani S., Boroujerdi M., Calcitriol Reverses Induced Expression of Efflux Proteins and Potentiates Cytotoxic Activity of Gemcitabine in Capan-2 Pancreatic Cancer Cells, Journal of Pharmacy & Pharmaceutical Sciences 20 (2017) 295. 10.18433/J37W7R. 28885916

[21] Yu W.-D., Ma Y., Flynn G., Muindi J.R., Kong R.-X., Trump D.L., Johnson C.S., Calcitriol enhances gemcitabine antitumor activity in vitro and in vivo by promoting apoptosis in a human pancreatic carcinoma model system, Cell Cycle 9 (2010) 3094–3101. 10.4161/cc.9.15.12381. PMC3040927 20699664

[22] Wei D., Wang L., Liu Y., Hafley M.A., Tan L., Lorenzi P.L., Yang P., Zuo X., Bresalier R.S., Activation of Vitamin D/VDR Signaling Reverses Gemcitabine Resistance of Pancreatic Cancer Cells Through Inhibition of MUC1 Expression, Dig Dis Sci 68 (2023) 3043–3058. 10.1007/s10620-023-07931-3. 37071246 PMC12289342

[23] K N S., Shetty P., Devaranagadi B., Hundekari I.A., Targeting ERβ1-Positive Triple-Negative Breast Cancer: Molecular Effects of Calcitriol and 17β-Estradiol, Cureus (2025). 10.7759/cureus.82934. PMC12103257 40416168

[24] Slominski A.T., Janjetovic Z., Fuller B.E., Zmijewski M.A., Tuckey R.C., Nguyen M.N., Sweatman T., Li W., Zjawiony J., Miller D., Chen T.C., Lozanski G., Holick M.F., Products of Vitamin D3 or 7-Dehydrocholesterol Metabolism by Cytochrome P450scc Show Anti-Leukemia Effects, Having Low or Absent Calcemic Activity, PLoS One 5 (2010) e9907. 10.1371/journal.pone.0009907. 20360850 PMC2845617

[25] Slominski A.T., Kim T.-K., Janjetovic Z., Tuckey R.C., Bieniek R., Yue J., Li W., Chen J., Nguyen M.N., Tang E.K.Y., Miller D., Chen T.C., Holick M., 20-Hydroxyvitamin D _2_ is a noncalcemic analog of vitamin D with potent antiproliferative and prodifferentiation activities in normal and malignant cells, American Journal of Physiology-Cell Physiology 300 (2011) C526–C541. 10.1152/ajpcell.00203.2010. 21160030 PMC3063966

[26] Wasiewicz T., Piotrowska A., Wierzbicka J., Slominski A.T., Zmijewski M.A., Antiproliferative Activity of Non-Calcemic Vitamin D Analogs on Human Melanoma Lines in Relation to VDR and PDIA3 Receptors, Int J Mol Sci 19 (2018) 2583. 10.3390/ijms19092583. 30200275 PMC6163194

[27] Slominski A., Brożyna A., Kim T.-K., Elsayed M., Janjetovic Z., Qayyum S., Slominski R., Oak A., Li C., Podgorska E., Li W., Jetten A., Tuckey R., Tang E., Elmets C., Athar M., CYP11A1-derived vitamin D hydroxyderivatives as candidates for therapy of basal and squamous cell carcinomas, Int J Oncol 61 (2022) 96. 10.3892/ijo.2022.5386. 35775377 PMC9262157

[28] Skobowiat C., Oak A.S.W., Kim T.-K., Yang C.H., Pfeffer L.M., Tuckey R.C., Slominski A.T., Noncalcemic 20-hydroxyvitamin D3 inhibits human melanoma growth in *in vitro* and *in vivo* models, Oncotarget 8 (2017) 9823–9834. 10.18632/oncotarget.14193. 28039464 PMC5354773

[29] Slominski A.T., Brożyna A.A., Skobowiat C., Zmijewski M.A., Kim T.-K., Janjetovic Z., Oak A.S., Jozwicki W., Jetten A.M., Mason R.S., Elmets C., Li W., Hoffman R.M., Tuckey R.C., On the role of classical and novel forms of vitamin D in melanoma progression and management, J Steroid Biochem Mol Biol 177 (2018) 159–170. 10.1016/j.jsbmb.2017.06.013. 28676457 PMC5748362

[30] Bhattacharjee V., Zhou Y., Yen T., A synthetic lethal screen identifies the Vitamin D receptor as a novel gemcitabine sensitizer in pancreatic cancer cells, Cell Cycle 13 (2014) 3839–3856. 10.4161/15384101.2014.967070. 25558828 PMC4615005

[31] Marcinkowska E., Gocek E., Heat shock protein 90 interacts with vitamin D receptor in human leukemia cells, J Steroid Biochem Mol Biol 121 (2010) 114–116. 10.1016/j.jsbmb.2010.01.013. 20138989

[32] Lutz W., Kohno K., Kumar R., The Role of Heat Shock Protein 70 in Vitamin D Receptor Function, Biochem Biophys Res Commun 282 (2001) 1211–1219. 10.1006/bbrc.2001.4711. 11302745

[33] Song C.W., Park H.J., Lee C.K., Griffin R., Implications of increased tumor blood flow and oxygenation caused by mild temperature hyperthermia in tumor treatment, International Journal of Hyperthermia 21 (2005) 761–767. 10.1080/02656730500204487. 16338859

[34] Frey B., Weiss E.-M., Rubner Y., Wunderlich R., Ott O.J., Sauer R., Fietkau R., Gaipl U.S., Old and new facts about hyperthermia-induced modulations of the immune system, International Journal of Hyperthermia 28 (2012) 528–542. 10.3109/02656736.2012.677933. 22690925

[35] Szyszka P., Zmijewski M.A., Slominski A.T., New vitamin D analogs as potential therapeutics in melanoma., Expert Rev Anticancer Ther 12 (2012) 585–99. 10.1586/era.12.40. 22594894 PMC3368500

[36] Bienia A., Wiecheć-Cudak O., Murzyn A.A., Krzykawska-Serda M., Photodynamic Therapy and Hyperthermia in Combination Treatment-Neglected Forces in the Fight against Cancer., Pharmaceutics 13 (2021). 10.3390/pharmaceutics13081147. PMC8399393 34452108

[37] Krzykawska-Serda M., Ho J.C.-S., Ware M.J., Law J.J., Newton J.M., Nguyen L., Agha M., Curley S.A., Corr S.J., Ultrasound Doppler as an Imaging Modality for Selection of Murine 4T1 Breast Tumors for Combination Radiofrequency Hyperthermia and Chemotherapy, Transl Oncol 11 (2018) 864–872. 10.1016/j.tranon.2018.04.010. 29763773 PMC6019683

[38] Hegyi G., Szigeti G.P., Szász A., Hyperthermia versus Oncothermia: Cellular Effects in Complementary Cancer Therapy, Evidence-Based Complementary and Alternative Medicine 2013 (2013) 1–12. 10.1155/2013/672873. PMC3638606 23662149

[39] Raoof M., Zhu C., Cisneros B.T., Liu H., Corr S.J., Wilson L.J., Curley S.A., Hyperthermia Inhibits Recombination Repair of Gemcitabine-Stalled Replication Forks, JNCI: Journal of the National Cancer Institute 106 (2014). 10.1093/jnci/dju183. PMC4155430 25128695

[40] He Q., Zheng Y., Lu L., Shen H., Gu W., Yang J., Zhang X., Jin H., Hyperthermia improves gemcitabine sensitivity of pancreatic cancer cells by suppressing the EFNA4/β-catenin axis and activating dCK, Heliyon 10 (2024) e28488. 10.1016/j.heliyon.2024.e28488. 38590861 PMC10999932

[41] Ahmed K., Tabuchi Y., Kondo T., Hyperthermia: an effective strategy to induce apoptosis in cancer cells, Apoptosis 20 (2015) 1411–1419. 10.1007/s10495-015-1168-3. 26354715

[42] Yang H., Xu S., Tang L., Gong J., Fang H., Wei J., Su D., Targeting of non-apoptotic cancer cell death mechanisms by quercetin: Implications in cancer therapy., Front Pharmacol 13 (2022) 1043056. 10.3389/fphar.2022.1043056. 36467088 PMC9708708

[43] Orth M., Metzger P., Gerum S., Mayerle J., Schneider G., Belka C., Schnurr M., Lauber K., Pancreatic ductal adenocarcinoma: biological hallmarks, current status, and future perspectives of combined modality treatment approaches, Radiation Oncology 14 (2019) 141. 10.1186/s13014-019-1345-6. 31395068 PMC6688256

[44] BLISS C.I., THE TOXICITY OF POISONS APPLIED JOINTLY1, Annals of Applied Biology 26 (1939) 585–615. 10.1111/j.1744-7348.1939.tb06990.x.

[45] Yang K.-L., Huang C.-C., Chi M.-S., Chiang H.-C., Wang Y.-S., Hsia C.-C., Andocs G., Wang H.-E., Chi K.-H., In vitro comparison of conventional hyperthermia and modulated electro-hyperthermia, Oncotarget 7 (2016) 84082–84092. 10.18632/oncotarget.11444. 27556507 PMC5356646

[46] Mao W., Li W., Hu X., Tumor hyperthermia research progress and application prospect in tumoroids (Review), Mol Clin Oncol 20 (2024) 31. 10.3892/mco.2024.2729. 38476334 PMC10928662

[47] Trump D.L., Deeb K.K., Johnson C.S., Vitamin D: Considerations in the Continued Development as an Agent for Cancer Prevention and Therapy, The Cancer Journal 16 (2010) 1–9. 10.1097/PPO.0b013e3181c51ee6. 20164683 PMC2857702

[48] Slominski A.T., Kim T.-K., Li W., Postlethwaite A., Tieu E.W., Tang E.K.Y., Tuckey R.C., Detection of novel CYP11A1-derived secosteroids in the human epidermis and serum and pig adrenal gland, Sci Rep 5 (2015) 14875. 10.1038/srep14875. 26445902 PMC4597207

[49] Kim T., Slominski R.M., Pyza E., Kleszczynski K., Tuckey R.C., Reiter R.J., Holick M.F., Slominski A.T., Evolutionary formation of melatonin and vitamin D in early life forms: insects take centre stage, Biological Reviews 99 (2024) 1772–1790. 10.1111/brv.13091. 38686544 PMC11368659

[50] Yadav B., Wennerberg K., Aittokallio T., Tang J., Searching for Drug Synergy in Complex Dose–Response Landscapes Using an Interaction Potency Model, Comput Struct Biotechnol J 13 (2015) 504–513. 10.1016/j.csbj.2015.09.001. 26949479 PMC4759128

